# A Bilayered Wood-Poly(3,4-ethylenedioxythiophene):Polystyrene Sulfonate Hydrogel Interfacial Evaporator for Sustainable Solar-Driven Sewage Purification and Desalination

**DOI:** 10.3390/nano13162321

**Published:** 2023-08-12

**Authors:** Xinye Xu, Qi Zhao, Qi Liu, Junxiao Qiu, Shutong Yuan, Zhixin Wu, Ruping Yang, Jie Cao, Lina Wang, Jingkun Xu, Baoyang Lu

**Affiliations:** 1 Jiangxi Key Lab of Flexible Electronics, Flexible Electronics Innovation Institute, Jiangxi Science and Technology Normal University, Nanchang 330013, China; xinye_xu2021@163.com (X.X.); zhaoq0108@163.com (Q.Z.); liuqi1903794147@163.com (Q.L.); q251039921181@163.com (J.Q.); wzx1169297427@163.com (Z.W.); caojie9905@163.com (J.C.); linawang_hn@163.com (L.W.); 2 School of Pharmacy, Jiangxi Science and Technology Normal University, Nanchang 330013, China; shutongyuancs@163.com (S.Y.); rpyang02@163.com (R.Y.); 3 Electronic Materials Research Laboratory, Key Laboratory of the Ministry of Education, International Center for Dielectric Research, Shaanxi Engineering Research Center of Advanced Energy Materials and Devices, School of Electronic Science and Engineering, Xi’an Jiaotong University, Xi’an 710049, China; 4 College of Chemistry and Molecular Engineering, Qingdao University of Science and Technology, Qingdao 266042, China

**Keywords:** solar water purification, interfacial evaporator, sewage purification and desalination, PEDOT:PSS hydrogel, wood

## Abstract

Solar-driven interfacial evaporation and purification is a promising solar energy conversion technology to produce clean water or solve water scarcity. Although wood-based photothermal materials have attracted particular interest in solar water purification and desalination due to their rapid water supply and great heat localization, challenges exist given their complicated processing methods and relatively poor stability. Herein, we propose a facile approach for fabricating a bilayered wood-poly(3,4-ethylenedioxythiophene):polystyrene sulfonate (wood-PEDOT:PSS) hydrogel interfacial evaporator by direct drop-casting and dry-annealing. Benefiting from the unique combined merits of the wood-PEDOT:PSS hydrogel evaporator, i.e., excellent light absorption (~99.9%) and efficient photothermal conversion of nanofibrous PEDOT:PSS and the strong hydrophilicity and fast water transport from wood, the as-fabricated bilayered wood-PEDOT:PSS hydrogel evaporator demonstrates a remarkably high evaporation rate (~1.47 kg m^−2^ h^−1^) and high energy efficiency (~75.76%) at 1 kW m^−2^. We further demonstrate the practical applications of such an evaporator for sewage purification and desalination, showing outstanding performance stability and partial salt barrier capability against a continuous 10-day test in simulated seawater and an ultrahigh ion removal rate of 99.9% for metal ion-containing sewage. The design and fabrication of such novel, efficient wood-based interfacial evaporators pave the way for large-scale applications in solar water purification.

## 1. Introduction

The scarcity of clean water has become one of the most critical global challenges on account of population growth, environmental pollution, and climate change [1,2,3]. Up to now, a tremendous number of feasible techniques have been extensively employed for high-efficiency clean water production such as photocatalysis [4,5,6,7] and solar-driven water evaporation [8,9,10]. In particular, the solar-driven interfacial evaporation technology represents a compelling avenue for environmentally friendly and cost-effective clean water production, requiring no additional power input and exhibiting immense potencies in sustainable sewage treatment and desalination, which has demonstrated considerable promise in addressing the pressing water scarcity crisis [11,12,13,14,15]. A solar-driven interfacial evaporator typically comprises essential components, including a solar absorber for efficient solar energy absorption and conversion into thermal energy, a water transport layer for timely water replenishment to maximize evaporation, and a thermal insulator to effectively mitigate thermal energy losses following the photothermal conversion [8,16]. Numerous high-efficiency solar evaporators have been extensively investigated and reported, encompassing carbon-based [6,17,18,19], polymers-based [20,21,22,23], semiconductor-based [24], and plasma nanoparticle-based [25,26] evaporators. However, the majority of these evaporators encounter challenges such as intricate fabrication procedures, high production costs, and compromised long-term stability, thereby impeding their practical implementation in seawater desalination and wastewater treatment. To address the aforementioned issues, there exists an imperative demand to develop solar evaporators that exhibit rapid water transport, wide-spectrum light absorption, and superior thermal insulation properties, as well as featuring simplified and cost-effective preparation techniques, alongside excellent long-term stability.

Wood-based solar evaporators have garnered significant attention as outstanding candidates for solar vapor generation, owing to their distinctive properties such as lower density, open microchannels, capillary-induced hydrophilicity, and low thermal conductivity [27,28,29,30]. Several methods have been developed to convert wood into photothermal materials, including surface carbonization [31,32,33] and coating techniques. Coating materials encompass plasma metals [4,26], carbon nanotubes [34], polydopamine [35], graphite [36], or black nanoparticles [37]. Despite significant progress in the preparation of wood-based photothermal materials, various obstacles remain. First, it is urgent to establish a simple and scalable approach for fabricating photothermal materials. Second, achieving superhydrophilicity in wood through carbonization or most inorganic coatings, while simultaneously establishing robust interfacial connections, poses irreversible damage to the light-absorbing interface. Once the light-absorbing layer of wood-based photothermal materials is compromised, its light absorption capability diminishes, and the water transport channels become blocked, resulting in the loss of solar steam generation performance. These limitations severely hinder the scalability and commercial applications of wood-based materials. To overcome these challenges, the development of novel wood-based photothermal conversion materials is essential.

**Figure 1:**
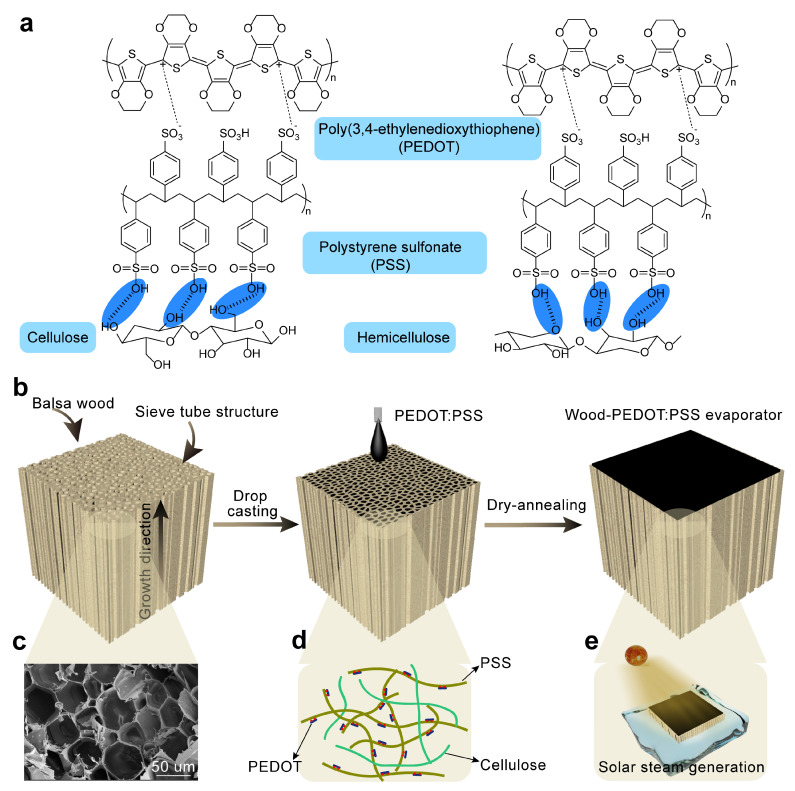
Design of the bilayered wood-PEDOT:PSS hydrogel evaporator. (**a**) Schematic illustration of the multimolecular interactions at the interconnection interface of PEDOT:PSS and wood. (**b**) Schematic illustration for the preparation of the bilayered wood-PEDOT:PSS hydrogel evaporator. (**c**) SEM image of the naturally porous structure of wood. (**d**) Schematic diagram of the PEDOT:PSS hydrogel network interaction with the fiber network on the wood surface. (**e**) The steam generation process of the wood-PEDOT:PSS hydrogel evaporator.

Inspired by the natural processes of plant transpiration and capillary action, we selected basswood, which has a lower density (compared with water) and exhibits superhydrophilicity induced by capillary action, as the water transport substrate. To enhance the localized heating on the wood surface, a light-absorbing and hydrophilic conjugated polymer PEDOT:PSS coating was deposited on the wood surface. Here we developed a bilayered wood-based interfacial evaporator with a stabilized nanofibrous PEDOT:PSS hydrogel coating through a simple methodology of drop casting and dry-annealing. The physical crosslinking network formed by the semi-crystalline domains rich in PEDOT and the hydrophilic matrix rich in PSS in nanofibrous PEDOT:PSS interacted with the typical hydrophilic network of cellulose fibers in wood (Figure 1a), thereby stabilizing the interconnection between the two interfaces. Similar to the moisture transport mechanism observed in trees, the wood-PEDOT:PSS hydrogel evaporator achieved water transport through capillary action, coupled with efficient photothermal conversion at the top surface and stable evaporation facilitated by intermolecular forces between the two components. The wood-PEDOT:PSS hydrogel evaporator featured effective broadband light absorption, ease of fabrication, and lightweight and intrinsic thermal insulation, as well as the natural water transportation channel, making it high-performance and highly competitive in solar-driven interfacial evaporation and desalination. As a consequence, this well-designed and high-efficiency bilayered wood-PEDOT:PSS hydrogel solar evaporator demonstrated remarkable light absorption (~99.9%), a high evaporation rate (~1.47 kg m^−2^ h^−1^), and commendable energy efficiency (~75.76%), surpassing the majority of reported wood solar evaporators. Therefore, the wood-PEDOT:PSS hydrogel solar evaporator holds great promise for various applications in solar evaporation and seawater desalination. In conclusion, this simplified and efficient fabrication method, with an outstanding evaporation performance and a stable structural design, offers a novel approach and strategy for the commercial application and large-scale production of wood-based evaporators. And it opens up broad prospects for the widespread utilization of solar-driven evaporation technology in applications such as water purification and desalination.

## 2. Materials and Methods

### 2.1. Materials

Poly(3,4-ethylenedioxythiophene):polystyrene sulfonate conductive particles (PEDOT:PSS) were obtained from Sigma-Aldrich (Shanghai, China). Natural Basha wood was obtained from Taobao. All other reagents were analytical grade and directly employed without further purification.

### 2.2. Fabrication of Wood-PEDOT:PSS Hydrogel Interfacial Evaporator

A piece of natural balsa wood was cut along its growth direction and placed with its lumen surface facing upward. Subsequently, 0.1 g liquid nitrogen freeze-dried PEDOT:PSS nanofibers were added to 9.9 mL DI water and well mixed with syringes, and then a sticky solution A was obtained. The wood surface was uniformly deposited with sticky solution A by drop-casting, with one, two, three, and four layers of coating applied sequentially, with each coating layer applied after drying at 30 °C. The coated wood blocks were dried at 60 °C for 24 h, then placed in a 130 °C oven for 30 min. Afterward, the samples were cooled for 5 min at room temperature. This drying and annealing cycle was repeated three times, and then the samples were immersed in deionized water. The resulting nanofibrous PEDOT:PSS hydrogel layer was firmly adhered to the mesoporous scaffold of the wood and could be utilized as an efficient broadband light-absorbing layer for solar steam generation.

### 2.3. Characterizations

The SEM images (Hitachi S4800, Tokyo, Japan) showcased the morphology and microstructure of the bilayered wood-PEDOT:PSS hydrogel interfacial evaporator. The concentrations of cations such as Na^+^, Mg^2+^, K^+^, Ca^2+^, Cu^2+^, Zn^2+^, Pb^2+^, and Ni^2+^ were tracked by an inductively coupled plasma-mass spectrometer (Agilent 7700ce, Santa Clara, CA, USA) upon dilution in 2% HNO_3_ to make the loaded ion concentration lower than 10 ppm. The contact angles of various wood-PEDOT:PSS hydrogels were tested on an optical contact angle measuring instrument (SDC-100, Guangdong, China). A UV-vis-NIR spectrometer (Toupu TP720, Tianjin, China) was utilized to conduct absorption and reflectance spectroscopy. The absorbance of light (*A*) was determined using the following equation:

*A* = *1* − *T* − *R* (1)

where *R* and *T* denote the reflectance and transmittance of varying wood-PEDOT:PSS hydrogel evaporators, respectively. An infrared camera was used to observe the temperature distributions of the samples (HIKMICRO TPH21Pro-3AQF, Guangdong, China).

### 2.4. Photothermal Performance Test

The ambient conditions for photothermal evaporation tests were maintained at a relative humidity of ~60% and a room temperature of 25 °C. The photothermal evaporation experiments were carried out using a solar simulator (Education Au-light Co., CEL-HXUV300-T3, Beijing, China) equipped with an AM 1.5G filter (1 kW m^−2^, 1 sun). The solar flux was measured by an automatic optical power meter (Education Au-light Co., CEL-NP2000-2A, Beijing, China). The weight loss of water was recorded by recording the weight of the samples by electronic balance every 5 min (Sartorius BAS223, Beijing, China). The surface temperature of the photothermal hydrogel was captured using an infrared camera.

## 3. Results and Discussion

### 3.1. Design of Bilayered Wood-PEDOT:PSS Hydrogel Interfacial Evaporator

We prepared a nanofibrous PEDOT:PSS hydrogel that could swell by dispersing the PEDOT:PSS solution and drying it through annealing [38]. Based on this, we proposed a simple and efficient method for controllingly coating wood with nanofibrous PEDOT:PSS hydrogel to better coordinate each material’s qualities. Initially, we vigorously mechanically mixed concentrated PEDOT:PSS nanofibers and directly drop-cast them onto the wood surface, allowing penetration into its pores and forming a preliminary stable bond with the wood surface and the internal fiber structure. To further bind the nanofibrous PEDOT:PSS hydrogel with the wood fibers and increase the coating’s durability, continual dry-annealing procedures were carried out. This ensured the formation of stable and uniform nanofibrous PEDOT:PSS hydrogel layers on the wood surface (Figure 1b). During the initial stages of preparation, the wood was subjected to scanning electron microscopy (SEM) imaging (Figure 1c), elucidating an organized porous structure with proficient water absorption and transport capabilities via capillary action. As the PEDOT:PSS solution coating was incrementally applied, the PEDOT:PSS hydrogel network effectively infiltrated and interacted within the wood matrix, augmenting the intertwining phenomenon between chains (Figure 1d), subsequently yielding a PEDOT:PSS hydrogel layer of distinct thickness, ensuring robust interfacial connections within the wood microstructure. The prepared bilayered wood-PEDOT:PSS hydrogel evaporator is depicted in Figure S1, illustrating its actual appearance. The outer surface of the evaporator exhibited a black color, indicating the presence of the PEDOT:PSS coating, whereas the internal regions predominantly retained their original coloration. Notably, the coating thickness after swelling was quantified to be ~0.165 mm (Figure S2). In our fabricated wood-PEDOT:PSS hydrogel evaporator, as the water was conveyed from the wood to the PEDOT:PSS hydrogel layer, the latter’s swelling propensity enabled swift water diffusion, leading to the generation of a thin water film. Through photothermal conversion, the water within the film experienced heating and evaporation, culminating in a highly proficient interfacial solar evaporation generation process (Figure 1e).

### 3.2. Optical, Photothermal Conversion, and Water Transport Properties of Wood-PEDOT:PSS Hydrogel Interfacial Evaporator

**Figure 2:**
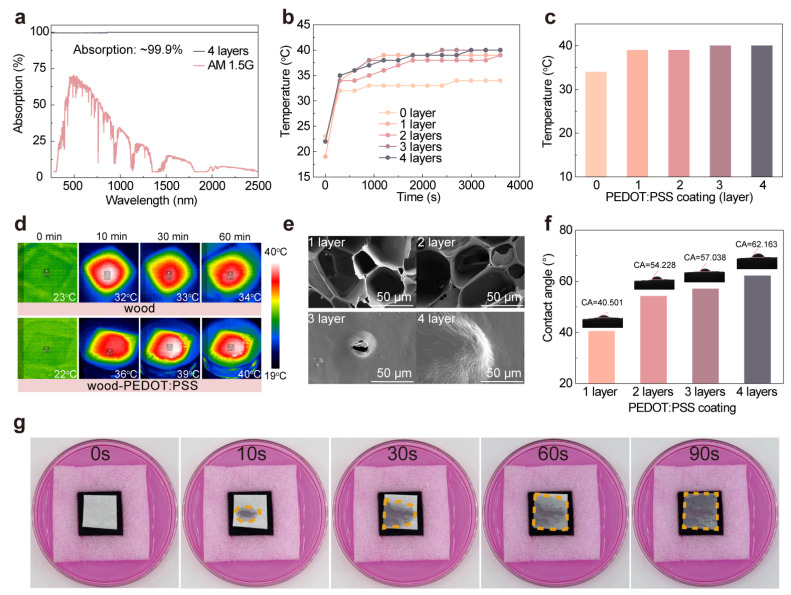
Optical, photothermal conversion, and water transport properties of the bilayered wood-PEDOT:PSS hydrogel evaporator. (**a**) Absorption spectra of nanofibrous PEDOT:PSS hydrogels. (**b**,**c**) Temperature variation (**b**) and maximum steady-state temperature (**c**) of wood and different wood-PEDOT:PSS hydrogel evaporators under 1 sun irradiation for one hour. (**d**) Infrared imaging of the surface temperature distribution of wood and wood-PEDOT:PSS hydrogel evaporator at 0, 10, 30, and 60 min solar irradiation. (**e**) SEM images of the wood-PEDOT:PSS evaporators with different nanofibrous PEDOT:PSS layers. (**f**) The contact angles of the wood-PEDOT:PSS hydrogel evaporators with different nanofibrous PEDOT:PSS hydrogel layers. Inset: photographs of the contact angle. (**g**) Water wettability of the bilayered wood-PEDOT:PSS hydrogel evaporator in Rhodamine B (RhB) solution.

In solar water evaporation, photothermal conversion materials typically require high broadband light absorption performance, excellent photothermal conversion efficiency, and rapid water transport capabilities. To investigate the light absorption capability of wood-PEDOT:PSS hydrogel evaporators, we performed light absorption tests on bilayered wood-based solar evaporators, which were coated with varying numbers of nanofibrous PEDOT:PSS hydrogel layers (from zero to four layers). These light absorption tests encompassed light absorption, light transmittance, and light reflectance. As the number of deposited layers of PEDOT:PSS hydrogel increased, the light absorption efficiency exhibited substantial enhancement, with respective values of ~99.80%, ~99.89%, ~99.90%, ~99.92%, and ~99.94% for each successive layer. Concurrently, the reflectance experienced a notable decrease, decreasing from ~0.17% to ~0.04%, and the light transmittance remained relatively constant (Figure S3 and Table 1). The findings demonstrated that all nanofibrous PEDOT:PSS hydrogels exhibited significant absorption across different spectral ranges. Specifically, the four-layer nanofibrous PEDOT:PSS hydrogels displayed outstanding light absorption (~99.9%) throughout the spectrum, with negligible reflectance and transmittance (Figure 2a and Table 1). These results suggested that the light absorption capacity of the bilayered wood-PEDOT:PSS hydrogel interfacial solar evaporator increased as the number of PEDOT:PSS hydrogel layers grew. However, deposition proved challenging when employing five-layer nanofibrous PEDOT:PSS hydrogels, possibly due to the incorporation of excessive hydrophobic PEDOT-enriched domains, leading to enhanced hydrophobicity and the consequent inability to form stable deposition layers. The achievement of efficient photothermal conversion capability was heavily dependent on the molecular thermal vibrations within the PEDOT:PSS material. To monitor the temperature distribution during solar-driven water evaporation, we employed infrared imaging techniques with the wood-PEDOT:PSS hydrogel evaporator. In Figure S4, infrared images were captured at different exposure times (0, 10, 30, and 60 min) for the nanofibrous PEDOT:PSS interfacial evaporator with varying layers. The results demonstrated that the surface temperature of the four-layer nanofibrous PEDOT:PSS hydrogel on the interfacial evaporator experienced a rapid increase within 10 min of solar irradiation, eventually reaching a steady-state equilibrium temperature of 40 °C after 40 min. This temperature was significantly higher than that observed for the pure wood interfacial evaporator without the deposition of nanofibrous PEDOT:PSS hydrogel (Figure 2b,d). Therefore, these findings indicated the effective achievement of a favorable thermal localization effect facilitated by utilizing the nanofibrous PEDOT:PSS hydrogel.

The water transport characteristics of evaporators played a crucial role in facilitating efficient interfacial evaporation, primarily relying on their physical structure encompassing porous channels and capillary transport, as well as the chemical structure governing the dynamic transfer and evaporation of hydrophobic water molecules. Natural wood possesses a porous structure and corresponding hydrophilic groups, which serve as a solid foundation for rapid water transport and the diffusion of water molecules. We evaluated the surface morphology of nanofibrous PEDOT:PSS hydrogels deposited on different layers of wood surfaces by SEM. The results revealed that natural wood exhibited a large and uniformly distributed vertically interconnected porous structure. Furthermore, it was observed that the deposition of the nanofibrous PEDOT:PSS hydrogel induced significant alterations in the morphologies and sizes of the pores and channels of the wood. With the increasing number of PEDOT:PSS hydrogel layers, the microstructure on the wood surface was nearly entirely covered (Figure 2e and Figure S5), resulting in the formation of a PEDOT:PSS hydrogel film with a certain thickness on top of the wood. Additionally, the combination of the PEDOT:PSS hydrogel film’s swelling ability and the capillary action of the wood contributed to the rapid transport of water and steam generation. As illustrated in Figure 2g, we placed a dry paper soaked in an organic dye solution on the surface of the bilayered solar evaporator and carefully observed the gradual wetting behavior of the paper. The experimental results clearly demonstrated that within a short duration of 90 s, the paper became nearly completely saturated, indicating the rapid water transport characteristics of the bilayered wood-PEDOT:PSS hydrogel solar evaporator and its capability to replenish water promptly to the evaporation surface. As the number of PEDOT:PSS hydrogel layers on the wood surface increased from one to four, we conducted contact angle tests on the surface of the bilayered wood-PEDOT:PSS hydrogel interfacial solar evaporator, observing an increase in the contact angle from 40.5° to 62.1° (Figure 2f). This further substantiated that the deposition of PEDOT:PSS had a certain impact on the water transport rate in the wood; however, it did not cause a complete loss of water transport capability on the wood surface. During this stage, the water transport mechanism shifted from capillary action on the wood surface to rapid diffusion within the hydrogel layer, thus achieving highly efficient solar steam generation.

### 3.3. Steam Generation Performances of the Wood-PEDOT:PSS Hydrogel Interfacial Evaporator

**Figure 3:**
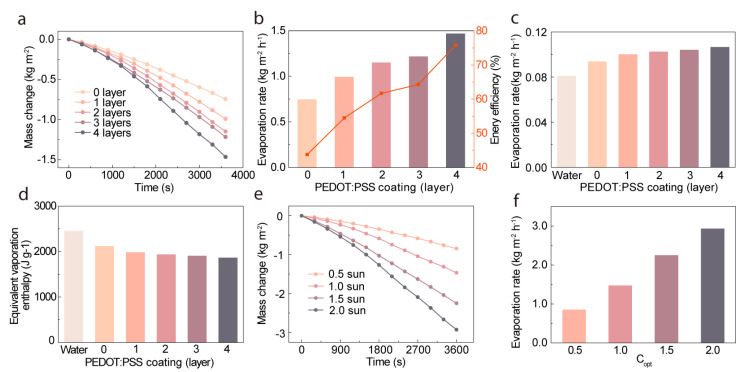
Evaporation performance of the wood-PEDOT:PSS hydrogel evaporator. (**a**) The mass change of water during solar evaporation for the wood-PEDOT:PSS evaporators with different nanofibrous PEDOT:PSS layers. (**b**) Evaporation rate and corresponding energy efficiency of different wood-PEDOT:PSS hydrogel evaporators. (**c**) Water evaporation rates under dark conditions. (**d**) Equivalent evaporation enthalpy for different wood-PEDOT:PSS hydrogel evaporators. (**e**,**f**) The mass change of water (**e**) and water evaporation rate (**f**) for four-layer nanofibrous PEDOT:PSS hydrogel under different solar irradiation intensities (0.5, 1.0, 1.5, and 2.0 sun).

In this research, a comprehensive quantification of the overall evaporation performance of the wood-PEDOT:PSS hydrogel evaporator was carried out, directly and systematically evaluating its evaporation rate and energy efficiency. The evaporation performance of the wood-PEDOT:PSS evaporator was assessed using the experimental evaporation test system, as depicted in Figure S7. The evaluation involved monitoring the mass change of water under 1 sun solar irradiation for 60 min. The results demonstrated that in the wood-PEDOT:PSS hydrogel evaporator with varying PEDOT:PSS layer numbers, the change in water mass exhibited a linear increase with increasing irradiation time (Figure 3b). Additionally, as the number of PEDOT:PSS layers increased from zero to four, the evaporation rate progressively rose, measuring at 0.74, 0.9, 1.15, 1.22, and 1.74 kg m^−2^ h^−1^. As illustrated in Figure 3c, the deposition of four layers of PEDOT:PSS hydrogel on the wood surface yielded the highest evaporation rate. Furthermore, scanning electron microscopy (SEM) was employed to characterize the surface structure of the evaporator following the evaporation process. As depicted in Figure S6, the results indicated no significant alteration in the surface structure, affirming the evaporator’s capability to maintain structural stability even under rigorous conditions of intense light irradiation and water immersion. The achievement of a high evaporation rate relied not only on the wood-PEDOT:PSS hydrogel evaporator’s rapid water transport, efficient water molecule diffusion, and effective photothermal conversion capabilities but also on the inherent energy requirements during the water evaporation process. The relatively low evaporative enthalpy of the hydrogel facilitated and promoted the water evaporation process. The equivalent evaporation enthalpy of water in the nanofibrous PEDOT:PSS hydrogel (${\Delta H}_{vap}$) is illustrated in Figure 3d and can be estimated by evaporating water with the same input power ($U_{in}$),

(2) $$U_{in}={\Delta H}_{vap}m_{0}={\Delta H}_{equ}m_{g}$$

In the equation, ${\Delta H}_{vap}$ represents the evaporation enthalpy of bulk water, $m_{0}$ denotes the mass change of bulk water (under dark conditions), and $m_{g}$ represents the mass change of different layers of PEDOT:PSS hydrogel coating of the evaporator during 1 h under dark conditions. As shown in Figure S7, the mass change tests conducted under dark conditions on bulk water bilayered wood-based evaporators with various PEDOT:PSS hydrogel coatings (from zero to four layers) revealed distinct values for the evaporation rates, namely, 0.081, 0.094, 0.1, 0.102, 0.104, and 0.106 kg m^−2^ h^−1^. The corresponding calculated values for the effective evaporation enthalpies were determined as 2450, 2115, 1979.6, 1933.2, 1903.5, and 1860.5 J g^−1^, respectively. These results demonstrated a significant reduction in the effective evaporation enthalpy of water within the nanofibrous PEDOT:PSS hydrogel compared to bulk water. Notably, the evaporator coated with four layers of PEDOT:PSS hydrogel exhibited a prominently decreased effective evaporation enthalpy, achieving an impressive low value of 1860.5 J g^−1^.

Energy efficiency (η) is calculated based on the corresponding $E_{equ}$ of the hydrogel, and in this case, (η) can be calculated using the following equation [39]:

(3) η=mEequCoptP0

Where *m* represents the mass flux at steady-state conditions, $E_{equ}$ is the equivalent evaporative enthalpy of water in the hydrogel, $P_{0}$ is the solar irradiation power of standard solar radiation (1 kW m^−2^), and $C_{opt}$ refers to the light concentration on the absorber surface. By optimizing the number of layers of PEDOT:PSS hydrogel (ranging from zero to four layers), the wood-PEDOT:PSS hydrogel evaporator achieved a substantial enhancement in energy efficiency under solar irradiation, increasing from ~43.73% to ~75.76% (Figure 3b). This notable evaporation performance surpassed the majority of conventional deposit light absorbers on wood surfaces (Table 2), and certain other classes of materials were favored, including carbon-based materials [17,31,32,34,36] plasmonic nanoparticles [40], and inorganic semiconductor-based materials [41], among others. The solar irradiance intensity had a significant impact on the evaporative performance of the hydrogel. As the solar irradiance intensity increased from a standard cloudy day illumination of 0.5 sun to a simulated concentrate sunlight condition of 2.0 sun, the curve depicting the mass change of water evaporated from the nanofibrous PEDOT:PSS hydrogel over time showed noticeable variations (Figure 3e). The evaporation rates also increased with the increasing light intensity, reaching 0.85, 1.47, 2.25, and 2.93 kg m^−2^ h^−1^, respectively (Figure 3f). This observation indicated that the increase in light intensity significantly promoted the evaporation process, thereby achieving higher evaporation rates and efficiency.

### 3.4. Applications in Sewage Purification of the Wood-PEDOT:PSS Hydrogel Interfacial Evaporator

**Figure 4:**
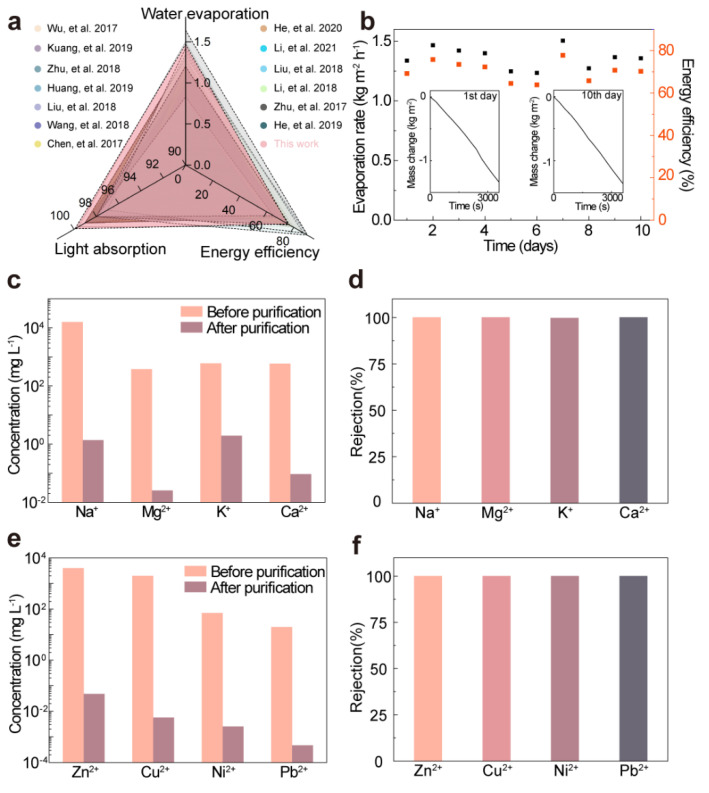
The stability and purification effect of the wood-PEDOT:PSS hydrogel evaporator. (**a**) Comparative performance of the wood-PEDOT:PSS hydrogel compared with other wood-based materials [18,19,25,31,32,33,34,35,36,37,42,43,44]. (**b**) Evaporation rates and corresponding energy efficiencies for four layers of wood-PEDOT:PSS hydrogel tested continuously for 10 days under one solar irradiation in a simulated seawater evaporation environment. (**c**,**d**) Metal ion concentrations (**c**) and corresponding ion removal rates (**d**) in different seawater samples before and after desalination. (**e**,**f**) Typical metal cation concentrations (**e**) and corresponding ion removal rates (**f**) in sewage before and after desalination.

As illustrated in Figure 4a, the distinct colored areas correspond to data values of various wood-based evaporators regarding light absorption, evaporation rate, and energy efficiency. The results are depicted that our developed wood-PEDOT:PSS hydrogel evaporator surpassed most of the previously reported advanced wood-based interfacial evaporators. The comparative analysis presented in Table 2 further substantiates its superior performance, outshining the majority of wood-based evaporators. These compelling findings position wood-PEDOT:PSS hydrogel as a leading contender for efficient and practical solar-driven water purification applications. Building upon the exemplary evaporation performance exhibited by the bilayered wood-PEDOT:PSS hydrogel evaporator as described above, it is imperative to investigate the long-term stability and durability of its performance. To evaluate the long-term stability of the wood-PEDOT:PSS hydrogel evaporator in a real-world environment, we conducted continuous monitoring of the evaporation mass change, evaporation rate, and corresponding energy efficiency for a duration of 10 days under 1 sun. During the experimental period, the wood-PEDOT:PSS hydrogel evaporator was immersed in simulated 3.5 wt.% seawater at night and exposed to natural sunlight. Astonishingly, after the 10-day testing period, the evaporation rate was determined to be ~1.358 kg m^−^^2^ h^−1^, exhibiting a marginal decrease of ~0.11 kg m^−^^2^ h^−1^ compared with the initial value of ~1.47 kg m^−^^2^ h^−1^. At the same time, the wood-PEDOT:PSS hydrogel evaporator also displayed consistent mass changes (Figure S8) and achieved a high average evaporation rate (~1.36 kg m^−2^ h^−1^) along with a corresponding average energy efficiency (~70.32%) (Figure 4b). These findings affirmed the outstanding reliability of the wood-PEDOT:PSS hydrogel evaporator, its enduring resilience to solar radiation, and its capacity for prolonged immersion in metal salt ion solutions.

The solar water purification capabilities of the bilayered wood-PEDOT:PSS hydrogel evaporator were investigated through its direct application in the desalination of natural seawater and treatment of simulated sewage. In order to evaluate the purification process of simulated seawater (3.5 wt.%) by the wood-PEDOT:PSS hydrogel evaporator, we employed ICP-MS to analyze the concentrations of major metal ions (e.g., Na^+^, Mg^2+^, K^+^, and Ca^2+^) in the seawater before and after purification. The outcomes revealed a significant reduction in the concentrations of the four major metal ions by approximately two to three orders of magnitude after purification using a wood-PEDOT:PSS hydrogel evaporator (Figure 4c). Additionally, each ion exhibited a removal rate exceeding 99.99% (Figure 4d), indicating the high efficiency and superiority desalination capabilities of the wood-PEDOT:PSS hydrogel evaporator. Concurrently, the accumulation of heavy metal ions in the human body can lead to enzyme and protein inactivation, thereby initiating chronic toxicity. By utilizing the wood-PEDOT:PSS hydrogel evaporator, we effectively eliminated heavy metal ions (e.g., Cu^2+^, Zn^2+^, Pb^2+^, and Ni^2+^) from the simulated sewage. The analysis of the purified water revealed a substantial reduction of heavy metal cation concentrations by four to six orders of magnitude before and after purification (Figure 4e), with a removal rate of heavy metal ions exceeding 99% (Figure 4f). As a result, the obtained results conclusively established the outstanding effectiveness of the wood-PEDOT:PSS hydrogel evaporator in addressing heavy metal pollution in wastewater.

## 4. Conclusions

This study used a simple and efficient method to develop a bilayered wood-based interfacial evaporator covered with nanofibrous PEDOT:PSS hydrogel for solar-driven sewage purification and desalination. PEDOT:PSS served as the light-to-heat conversion layer, enabling excellent broadband light absorption and efficient photothermal conversion. The wood acted as the water transport and thermal insulation layer, facilitating rapid water replenishment during the evaporation process and minimizing energy loss. The synergistic benefits of these features resulted in the bilayered wood-PEDOT:PSS hydrogel evaporator achieving high light absorption of ~99.9% and an impressive evaporation rate of ~1.47 kg m^−2^ h^−1^, with an energy conversion efficiency of ~75.76%. Moreover, even after prolonged exposure to sunlight and during saltwater evaporation experiments, the wood-PEDOT:PSS hydrogel evaporator exhibited superior evaporation performance compared with most reported wood-based interfacial evaporators. Additionally, it demonstrated remarkable durability and long-lasting salt rejection capabilities, achieving an impressive 99.9% efficiency in removing heavy metal ions from sewage. In conclusion, the design and fabrication method demonstrated in this research offer a new and promising strategy for environmentally friendly and cost-effective solar-driven interfacial evaporation and seawater desalination technologies, holding great potential to alleviate and address global water resource scarcity and environmental pollution challenges.

## Figures and Tables

**Table 1:** Absorptance, transmittance, and reflectance spectra of the PEDOT:PSS hydrogels with different layers in the wavelength range of 250–2500 nm.

PEDOT:PSS Hydrogel	Absorption (%)	Reflectance (%)	Transmittance (%)
zero-layer	99.80	0.17	0.03
one-layer	99.89	0.09	0.02
two-layer	99.90	0.08	0.02
three-layer	99.92	0.06	0.02
four-layer	99.94	0.04	0.02

**Table 2:** Performance comparison of wood-PEDOT:PSS hydrogel with current wood-based and other photothermal conversion materials.

Materials	Light Absorption (%)	Evaporation Rate (kg m^−2^ h^−1^)	Energy Efficiency (%)	References
Wood-PDA	90	1.38	87	[19]
Drilling holes	98	1.04	75.1	[32]
Plasmonic wood	99	1.0	68	[25]
Carbonized wood	99	1.3	57.3	[31]
C-L wood	96	1.08	74	[18]
CNT-coated tree	95	1.0	67.8	[42]
graphite coated wood	95	1.2	80	[36]
CuFeSe_2_ (NPs) decorated wood	99	1.3	67.7	[37]
carbonized bimodal evaporator	97	0.8	57	[33]
poplar-TA-Fe^3+^	98	1.34	90	[43]
Wood/Fe_2_O_3_/CNT	97	1.42	87.2	[44]
F wood/CNTs	98	0.83	65	[34]
PNPG wood	90	1.64	90.4	[35]
NP-Cu film	/	1.47	92.9	[45]
PG-10	95	1.42	96.6	[46]
TiN NPs	/	1.34	84.5	[47]
WTe_2_ nanosheet	/	1.09	74.8	[41]
Wood-PEDOT:PSS hydrogel	99.9	1.47	75.76	This work

## Data Availability

The data supporting this study’s findings are available from the corresponding author upon request.

## Supplementary Materials

The following supporting information can be downloaded at https://www.mdpi.com/article/10.3390/nano13162321/s1: Figure S1. Photographs of the pure wood evaporator and the bilayered wood-PEDOT:PSS hydrogel evaporator; Figure S2. Photographs of wood sections with four layers of PEDOT:PSS hydrogel both before and after the coating, as well as following hydrogel swelling; Figure S3. UV-vis-NIR absorption spectra in the wavelength range of 250–2500 nm. Inset: reflection spectra; Figure S4. IR imaging of the surface temperature distribution for nanofibrous PEDOT:PSS hydrogels under 1 sun irradiation time at 0, 10, 30, and 60 min; Figure S5. SEM images of the bilayered wood-PEDOT:PSS hydrogel evaporator at different magnifications; Figure S6. SEM images of the bilayered wood-PEDOT:PSS hydrogel interfacial evaporator before and after evaporating; Figure S7. The schematic diagram of the evaporation test; Figure S8. Stability testing of the wood-PEDOT:PSS hydrogel interfacial evaporator for 10 days.
