# Supplementary material for: A Bilayered Wood-Poly(3,4-ethylenedioxythiophene):Polystyrene Sulfonate Hydrogel Interfacial Evaporator for Sustainable Solar-Driven Sewage Purification and Desalination
Source: Nanomaterials (Basel). 2023 Aug 12;13(16):2321. doi: 10.3390/nano13162321 (PMC10457931; doi:10.3390/nano13162321)
Supplement: Supplementary file 1 [file nanomaterials-13-02321-s001.zip › nanomaterials-2507102-supplementary.pdf]

Supplementary information

Contents

Figure S1. Photographs of the pure wood evaporator and the bilayered wood-PEDOT:PSS hydrogel evaporator.

Figure S2. Photographs of wood sections with four-layer of PEDOT:PSS hydrogel both before and after the coating, as well as following hydrogel swelling.

Figure S3. UV-vis-NIR absorption spectra in the wavelength range of 250-2500 nm. Inset: reflection spectra.

Figure S4. IR imaging of the surface temperature distribution for PEDOT:PSS hydrogels under one sun irradiation time at 0, 10, 30, and 60 min.

Figure S5. SEM images of the bilayered wood-PEDOT:PSS hydrogel evaporator at different magnifications.

Figure S6. SEM images of the bilayered wood-PEDOT:PSS hydrogel interfacial evaporator before and after evaporating.

Figure S7. The schematic diagram of the evaporation test.

Figure S8. Stability testing of wood-PEDOT:PSS hydrogel interfacial evaporator for 10 days.

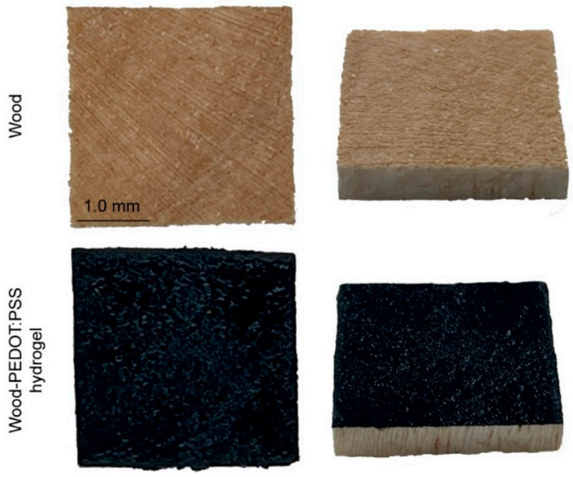

Figure S1. Photographs of the pure wood evaporator and the bilayered wood-PEDOT:PSS hydrogel evaporator.

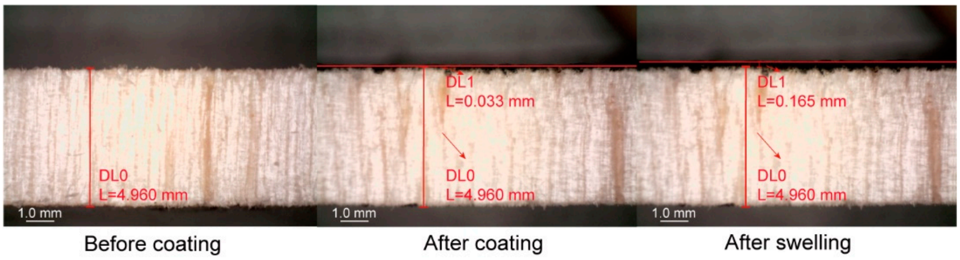

Figure S2. Photographs of wood sections with 4 layers of PEDOT:PSS hydrogel both before and after the coating, as well as following hydrogel swelling.

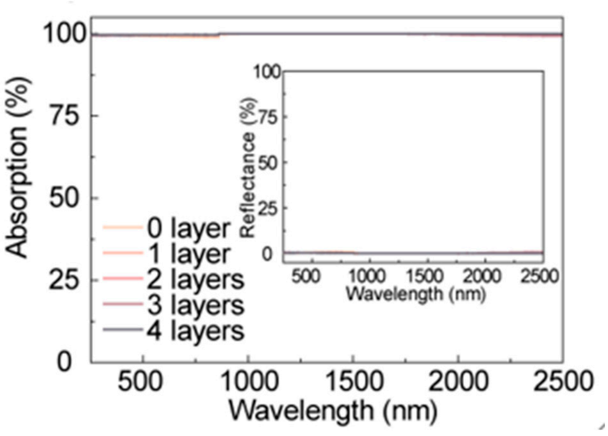

Figure S3. UV-vis-NIR absorption spectra in the wavelength range of 250-2500 nm. Inset: reflection spectra.

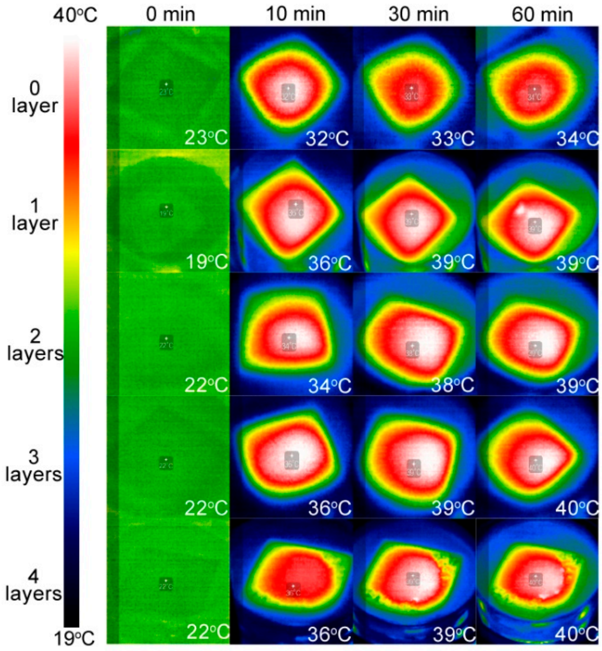

Figure S4. IR imaging of the surface temperature distribution for PEDOT:PSS hydrogels under one sun irradiation time at 0, 10, 30, and 60 min.

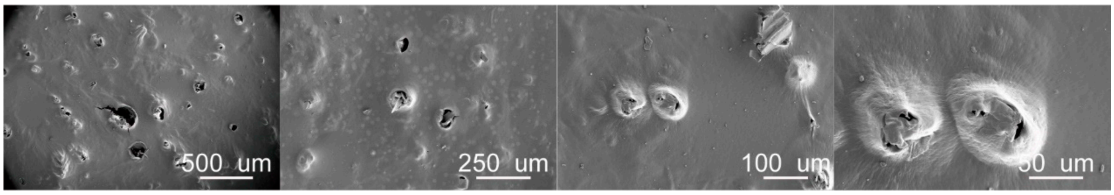

Figure S5. SEM images of the bilayered wood-PEDOT:PSS hydrogel evaporator at different magnifications.

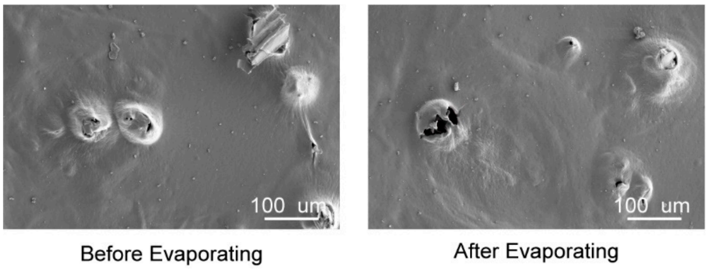

Figure S6. SEM images of the bilayered wood-PEDOT:PSS hydrogel interfacial evaporator before and after evaporating.

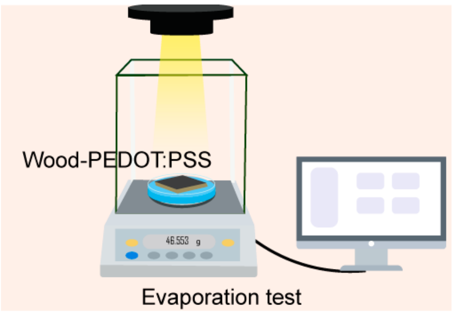

Figure S7. The schematic diagram of the evaporation test.

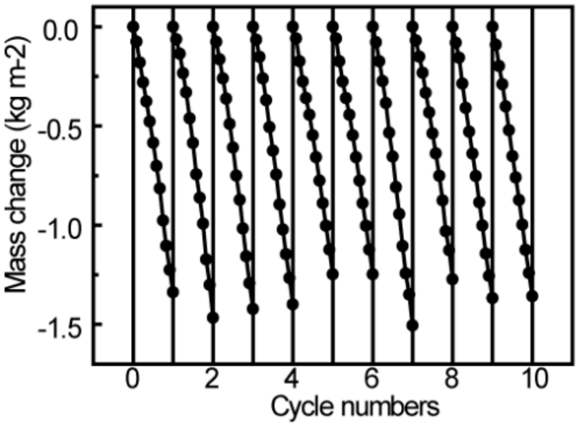

Figure S8. Stability testing of wood-PEDOT:PSS hydrogel solar evaporator for 10 days.

Long-term reliability and stability of the bilayered wood-PEDOT:PSS hydrogel evaporator via 10-day continuous monitoring: The bilayered wood-PEDOT:PSS hydrogel evaporator was placed on the surface of the simulated seawater outdoors from 11 pm to 7 am (overnight), and then the steam generation performance was tested under simulated solar irradiation of 1 kW m^{-2} for 1 h (typically from 8 pm to 9 pm). After the test, the system was again disposed of outdoors for the following tests. From the results (Figure 4b and S8), the evaporation rate was determined to be ~ 1.358 kg m^{-2} h^{-1}, exhibiting a marginal decrease of ~ 0.11 kg m^{-2} h^{-1} compared to the initial value of ~ 1.47 kg m^{-2} h^{-1}. Additionally, the average vapor generation rate was approximately ~1.36 kg m^{-2} h^{-1}, corresponding to an average energy efficiency of ~70.32%.
